# Notch–Sox9 Axis Mediates Hepatocyte Dedifferentiation in *Kras^G12V^*-Induced Zebrafish Hepatocellular Carcinoma

**DOI:** 10.3390/ijms23094705

**Published:** 2022-04-24

**Authors:** Junhui Sun, Qi Chen, Jianlong Ma

**Affiliations:** Institute of Developmental Biology and Regenerative Medicine, Southwest University, Beibei, Chongqing 400715, China; sjh2014@email.swu.edu.cn (J.S.); chenqi5231@email.swu.edu.cn (Q.C.)

**Keywords:** hepatocellular carcinoma, cancer model, dedifferentiation, Notch, Sox9

## Abstract

Liver cancer is one of the most prevalent cancers in humans. Hepatocytes normally undergo dedifferentiation after the onset of hepatocellular carcinoma, which in turn facilitates the progression of cancer. Although the process of hepatocellular carcinoma dedifferentiation is of significant research and clinical value, the cellular and molecular mechanisms underlying it are still not fully characterized. We constructed a zebrafish liver cancer model based on overexpression of the oncogene *kras^G12V^* to investigate the hepatocyte dedifferentiation in hepatocellular carcinoma. We found that, after hepatocarcinogenesis, hepatocytes dedifferentiated and the Notch signaling pathway was upregulated in this progress. Furthermore, we found that inhibition of the Notch signaling pathway or deficiency of *sox9b* both prevented hepatocyte dedifferentiation following hepatocellular carcinoma induction, reducing cancer metastasis and improving survival. In conclusion, we found that hepatocytes undergo dedifferentiation after hepatocarcinogenesis, a process that requires Notch signaling and likewise the activation of Sox9.

## 1. Introduction

Primary liver cancer is the sixth most commonly diagnosed cancer and the third leading cause of cancer deaths worldwide as of 2020, with approximately 906,000 new cases and 830,000 deaths. Primary liver cancer includes hepatocellular carcinoma (HCC) (75%-85% of cases) and intrahepatic cholangiocarcinoma (10–15%), as well as other rare types [1]. The general lack of regular cancer screening in the vast majority of developing countries has led to liver cancer often being diagnosed at advanced stages [2]. The main risk factors for HCC are chronic infection with hepatitis B virus (HBV) or hepatitis C virus (HCV), aflatoxin-contaminated food, excessive alcohol abuse, excess weight, type 2 diabetes, and smoking, with major risk factors varying by region [3]. Nonalcoholic fatty liver disease seems to have become increasingly common in recent years and has been noted as one of the reasons for the recurrence of liver cancer incidence in some regions [1]. Therefore, liver cancer remains a serious threat to public health, and there are still many issues to be addressed in the incidence, progression, and treatment of liver cancer. Further research on liver cancer by additional cancer models may shed light on a variety of issues. Because of their small embryos, quick growth cycle, and low cost of keeping, zebrafish are quite a suitable choice to build cancer models. They may aid in building a better understanding of the influence of differentiation in liver cancer progression, as well as the clinical translation of innovative medications and the expansion of the array of effective differentiation-based cancer therapies.

Previous studies have identified mature hepatocytes as the cells of origin of HCC [3]. These cells dedifferentiate into hepatocyte progenitor cells, which then become hepatocellular carcinoma cells that begin to express progenitor cell markers [4,5]. The clinical therapy of hepatocellular carcinoma has revealed that HCC patients with poorly differentiated tumors have a worse prognosis [6]. Differentiation therapy, which aims to induce and reverse tumor dedifferentiation, is predicted to be a potential therapeutic strategy. Dedifferentiation has been shown to be a phase in the advancement of hepatocellular carcinoma in previous investigations. As a consequence, efforts to restore the liver’s well-differentiated state may be an effective method to alleviate the poor prognosis associated with hepatocellular carcinoma treatment [7,8]. Therefore, it is necessary to investigate the factors that alter the expression levels during hepatocyte dedifferentiation. We may be able to uncover more targets for differentiation therapy by investigating the changes and roles of these variables in hepatocarcinogenesis and progression.

The Notch signaling pathway is a highly conserved pathway during development. This pathway is essential for biliary cell coordination and tubule formation [9]. The structure, homeostasis, and carcinogenesis of the liver are dependent on the Notch cascade [10]. The Notch cascade reaction is activated by direct cell–cell interactions followed by cleavage of the Notch receptor extracellular domain (NECD) [11]. This structural change leads to the release of the Notch intracellular structural domain (NICD), which translocates to the nucleus and recruits coactivators, such as Mastermind-like proteins (MAML1, MAML2, or MAML3), to form a transcriptional complex responsible for the induction of Notch target genes [11]. Although there is mounting evidence that Notch signaling is involved in a variety of cancers [12,13], the relevance of Notch signaling in hepatocellular carcinoma remains controversial and its particular function is not well understood. For example, some studies have shown that expression of the constitutively active form of Notch1 has an inhibitory effect on hepatocellular carcinoma cells and that blocking Notch with a γ-secretase inhibitor (GSI) in mice promotes the progression of hepatocellular carcinoma, suggesting that Notch operates as a tumor suppressor [14,15]. However, other studies have shown that the Notch signaling pathway drives HCC tumor growth and that inhibition of this pathway has antitumor effects [16]. Recently, several studies have revealed that the Notch signaling pathway acts as a driver of liver tumors [17,18,19]. Recent studies in mouse models suggest that different isoforms of Notch may have different roles in different models of hepatocellular carcinoma, and Notch may be pleiotropic, as a hepatocellular carcinoma driver and suppressor [20,21]. Although there are many reports on the Notch signaling pathway in hepatocellular carcinoma, the mechanism of Notch for hepatocarcinogenesis and cell migration is still not well clarified. Further study on Notch’s effect on hepatocyte dedifferentiation after hepatocarcinogenesis is important to point us in the proper direction for hepatocellular carcinoma treatment.

## 2. Results

### 2.1. Induction of Hepatocellular Carcinoma by DOX-Activated Tet-On System in Zebrafish

**Figure 1:**
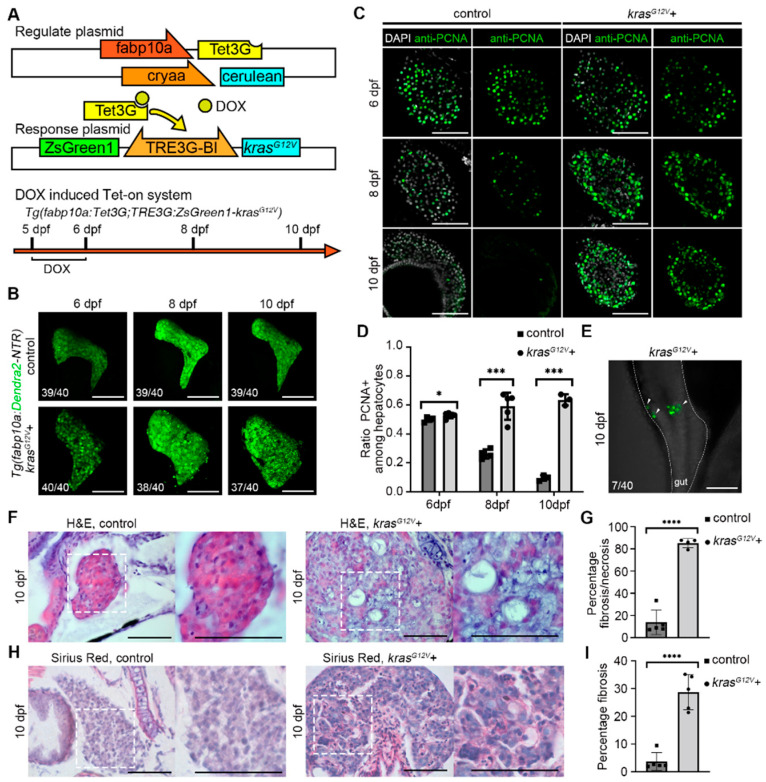
Induction of *kras^G12V^* overexpression in zebrafish liver cancer by Tet-On system. (**A**) Components of the Tet-On system and doxycycline (DOX) treatment strategy for induction of liver cancer. (**B**) Confocal images showed zebrafish liver after DOX activation of *kras^G12V^* from 6 days postfertilization (dpf) to 10 dpf. (**C**,**D**) antibody staining results of proliferating cell nuclear antigen (PCNA) in the control group (*n* = 11) and *kras^G12V^*+ group (*n* = 13). (**E**) Confocal images showed the presence of a green, fluorescent signal in the intestine outside the liver after DOX activation of *kras^G12V^*. (**F**) H&E staining to confirm the morphology of the liver in the control and *kras^G12V^*+ groups. (**G**) Statistics of the percentage fibrosis/necrosis area in the liver in the control (*n* = 5) and *kras^G12V^*+ (*n* = 4) groups. (**H**) Sirius Red staining of the liver in the control and *kras^G12V^*+ groups. (**I**) Statistics of the percentage fibrosis area in the liver in the control (*n* = 5) and *kras^G12V^*+ (*n* = 5) groups. Numbers indicate the percentage of larvae exhibiting this expression. Asterisks show significance: *—*p* < 0.05; ***—*p* < 0.001; ****—*p* < 0.0001. Scale bars—100 μm; error bars—S.D.

Several methods have been used to artificially induce hepatocellular carcinoma in mice and, by extension, in zebrafish [22,23]. Based on the previously reported results, we constructed a zebrafish model of liver cancer using the new generation Tet-On 3G system with mutated *kras^G12V^* as the oncogene [24,25]. The new generation Tet-On 3G has a marked improvement over the previous system with a significant reduction in basal expression and an increased sensitivity to doxycycline (DOX) [26]. Tet-On 3G systems have lower basal expression and higher sensitivity to the doxycycline (Dox) inducer than their predecessors. When compared with the previous tightest promoter, the pTRE3G promoter contains mutations that reduce background expression by 5–20-fold. Mutations in the Tet-On 3G transactivator greatly increase its sensitivity to Dox. This improved sensitivity is especially beneficial for in vivo studies in tissues where high Dox concentrations are difficult to achieve [24]. Previous studies have reported that DOX, the inducer of the Tet-On system, is devoid of noticeable toxicity, has minimal effects on the hepatic transcriptome, and does not alter the overall characteristics of hepatocytes [27]. We constructed a transgenic strain *Tg(fabp10a:Tet3G;TRE3G:kras^G12V^-ZsGreen1)^cq136^* with liver-specific overexpression of the oncogene *kras^G12V^* in zebrafish using the DOX-activated Tet-On system. The DOX-treated *Tg(fabp10a:Dendra2-NTR)^cq1^* was termed as the control group. The oncogene *kras^G12V^*, overexpressed in *Tg(fabp10a:Tet3G;TRE3G:kras^G12V^-ZsGreen1)^cq136^* (*kras^G12V^*+ group) by DOX treatment (Figure 1A), and the liver of zebrafish in the *kras^G12V^*+ group was significantly enlarged compared with the control group after DOX treatment (Figure 1B and Figure S1A). Meanwhile, DOX caused no detectable effect in the control group.

Immunofluorescence staining of proliferating cell nuclear antigen (PCNA), a marker of cell proliferation expressed in the nuclei of cells during the DNA synthesis phase of the cell cycle [28], showed that the proportion of proliferating hepatocytes in the control group gradually decreased with the end of liver development, while the proportion of proliferating hepatocytes in the *kras^G12V^*+ group gradually increased (Figure 1C,D). At 10 days postfertilization (dpf), certain larvae showed the presence of induced hepatocytes in the intestine outside the liver, indicating the migration of hepatocellular carcinoma cells (Figure 1E). Meanwhile, we observed a green flow of hepatocytes in the blood vessels in these larvae (results not presented). The hematoxylin–eosin (H&E) staining revealed a significant histological change in the liver in the *kras^G12V^*+ group, and the loss of eosin in the *kras^G12V^*+ group indicates the fibrosis or necrosis (Figure 1F,G). Notice that due to the enlargement of the liver after carcinogenesis, the entire field of the right panel view was filled with the liver tissue. The Sirius Red staining showed fibrosis occurred in the *kras^G12V^*+ group (Figure 1H,I). From the above experiments, we concluded that specific overexpression of the protooncogene *kras^G12V^* in zebrafish by the DOX-induced Tet-On system lead to hepatocytes’ over proliferation, cell migration, and fibrosis, and these indicated that hepatocellular carcinoma was successfully induced in zebrafish.

### 2.2. Hepatocyte Dedifferentiation Occurred after Hepatocarcinogenesis

**Figure 2:**
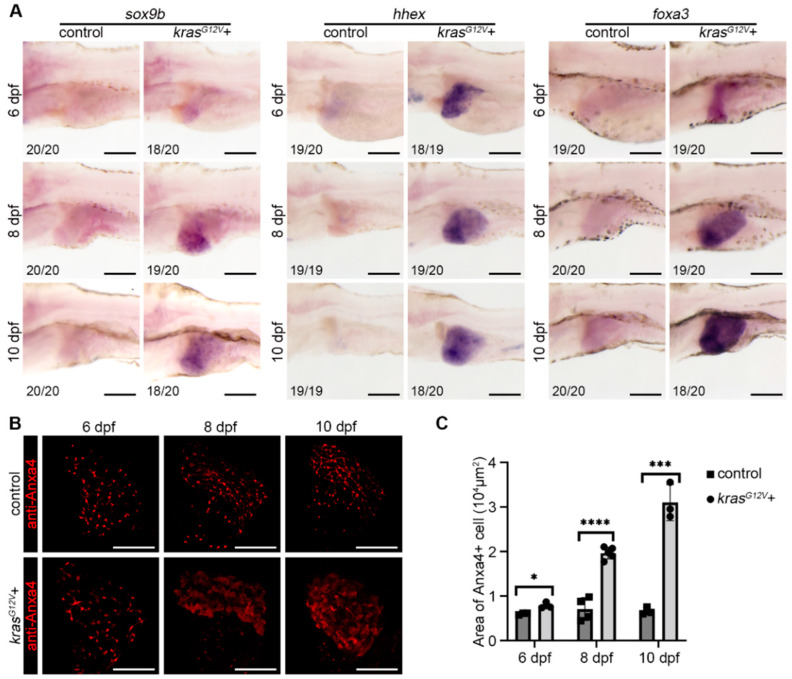
Zebrafish hepatocytes undergo dedifferentiation and biliary duct activation after *kras^G12V^* induction. (**A**) Whole-mount in situ hybridization (WISH) results showed the expression pattern of *sox9b*, *hhex*, and *foxa3* at 6 dpf, 8 dpf, and 10 dpf, respectively, after DOX activation of *kras^G12V^*. (**B**,**C**) Antibody staining of biliary duct marker Anxa4 in zebrafish liver expression at 6 dpf, 8 dpf, and 10 dpf after DOX induction, and statistics of Anxa4+ area in the liver in the control (*n* = 10) and *kras^G12V^*+ (*n* = 11) groups. Numbers indicate the percentage of larvae exhibiting this expression. Asterisks show significance: *—*p* < 0.05; ***—*p* < 0.001; ****—*p* < 0.0001. Scale bars—100 μm; error bars—S.D.

Cell fate in the liver is altered with hepatocarcinogenesis and, usually, biliary duct cells, and hepatocytes are drastically altered after hepatocarcinogenesis [3]. To investigate the fate of hepatocytes and biliary duct cells after hepatocarcinogenesis, we used whole-mount in situ hybridization (WISH) to detect hepatic progenitor cell marker genes after hepatocarcinogenesis and antibody staining to detect changes in biliary duct morphology. Previously, our group reported that liver progenitor cells express *hhex*, *foxa3*, and *sox9b* genes during liver regeneration [29]. WISH of the liver progenitor marker genes *hhex* and *foxa3*, and the biliary duct marker gene *sox9b* showed that *hhex*, *foxa3*, and *sox9b* expression was upregulated after hepatocarcinogenesis, indicating the hepatocytes exhibited a progenitor characteristic (Figure 2A). EMT is important in cancer progression, and E-Cadherin (CDH1) appears to be downregulated during EMT [30]. We detected CDH1 expression in the liver by antibody staining after hepatocarcinogenesis. In the control group, CDH1 signaling was expressed intensely in the biliary duct cells (Figure S1B). Meanwhile, in the *kras^G12V^*+ group, the expression pattern of CDH1 was disturbed, the biliary ducts expressing CDH1 thickened at 8 dpf, the biliary duct shape was almost lost by 10 dpf, and the expression of CDH1 in the liver was downregulated (Figure S1B). A previous article reported that Anxa4, as a biliary duct marker, also marks liver progenitor cells and had been considered as a marker of liver dedifferentiation [29,31]. Biliary duct antibody staining (anti-Anxa4) showed that the biliary ducts in the *kras^G12V^*+ group thickened after hepatocarcinogenesis, and the whole liver expressed biliary duct markers by 10 dpf (Figure 2B,C). From the above experiments, we concluded that, after hepatocarcinogenesis, the biliary duct activation occurred and hepatocytes in the liver undergo dedifferentiation along with the expression of biliary duct markers.

### 2.3. Hepatocyte Dedifferentiation was Suppressed by Inhibition of Notch Signaling after Hepatocarcinogenesis

**Figure 3:**
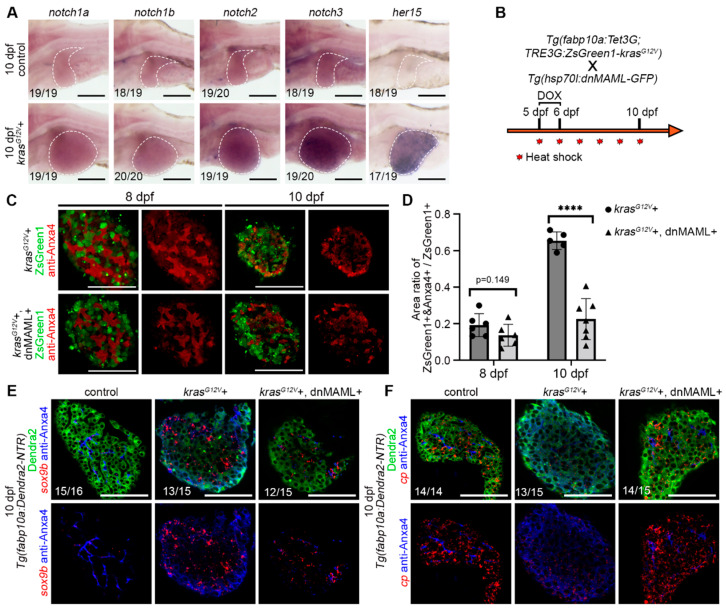
The Notch signaling pathway was upregulated after hepatocellular carcinoma induction, and the inhibition of Notch signaling suppressed hepatocyte dedifferentiation. (**A**) WISH results showed the expression pattern of *notch1a*, *notch1b*, *notch2*, *notch3*, and *her15* at 10 dpf after DOX activation of *kras^G12V^*. (**B**) *Tg(fabp10a:Tet3G;TRE3G:kras^G12V^-ZsGreen1)* with *Tg(Hsp70l:dnMAML-GFP)* double transgenic fish line treatment strategy. (**C**) Monolayer images of Anxa4 antibody staining showed expression changes in the DOX-induced *kras^G12V^*+ and *kras^G12V^*+ and dnMAML+ groups at 8 dpf and 10 dpf. (**D**) Statistics of Anxa4 + and ZsGreen1 + /ZsGreen1+ ratio in the DOX-induced *kras^G12V^*+ (*n* = 11) and *kras^G12V^*+ and dnMAML+ (*n* = 13) groups at 8 dpf and 10 dpf. (**E**) The results of fluorescent in situ hybridization (FISH) showed *sox9b* expression in the DOX-induced control and *kras^G12V^*+ and *kras^G12V^*+ and dnMAML+ groups at 10 dpf. (**F**) The results of FISH showed *cp* expression in the DOX-induced control, *kras^G12V^*+ and *kras^G12V^*+ and dnMAML+ groups at 10 dpf. Numbers indicate the proportion of larvae exhibiting that expression. Asterisks show significance: ****—*p* < 0.0001. Scale bars—100 μm; error bars—S.D.

The Notch signaling pathway plays an important role in liver development and regeneration [11]. In liver regeneration, Notch mediates dedifferentiation of biliary duct, and liver sinusoidal endothelial cells had been reported [29,32,33]. Therefore, we checked the expression of Notch receptors (*notch1a*, *notch1b*, *notch2*, and *notch3*), as well as the Notch target gene *her15* in the control and *kras^G12V^*+ group (Figure 3A). The Notch signaling pathway was upregulated after DOX induction (Figure 3A). Therefore, we use the dominant-negative isoform of murine Mastermind-like (dnMAML) protein to inhibit the Notch signaling pathway after hepatocarcinogenesis (Figure 3B). The dnMAML is a truncated form of MAML1 retaining an N-terminal interaction domain but lacking a C-terminal act as dominant-negative inhibitors of the intracellular domain of Notch function [34]. The dnMAML binds to the Notch transcriptional complex but lacks the activity to recruit essential cofactors, thereby blocking Notch activity, already applied and shown to work on the Notch pathway in zebrafish in some work [35,36]. The Dox-treated *Tg(fabp10a:Tet3G;TRE3G:kras^G12V^-ZsGreen1)^cq136^* (*kras^G12V^*+ group) and *Tg(fabp10a:Tet3G;TRE3G:kras^G12V^-ZsGreen1)^cq136^ X Tg(Hsp70l:dnMAML-GFP)* (*kras^G12V^*+ and dnMAML+ group) were heat-shocked simultaneously (Figure 3B). By inhibiting the Notch signaling pathway, we found that the morphological changes of biliary ducts after hepatocarcinogenesis were not completely inhibited, but the expression of biliary duct markers in hepatocytes was suppressed (Figure 3C,D). Meanwhile, fluorescent in situ hybridization (FISH) of biliary duct marker gene *sox9b* showed that *sox9b* expression was upregulated in hepatocytes after hepatocarcinogenesis, while the expression of *sox9b* exhibited no upregulation in hepatocytes when Notch signaling pathway was inhibited (Figure 3E). FISH of the liver functional marker gene *cp* showed that *cp* expression was downregulated in hepatocytes after hepatocarcinogenesis and no downregulation of *cp* expression in hepatocytes occurred when the Notch signaling pathway was inhibited (Figure 3F). Therefore, we concluded that the Notch signaling pathway is upregulated after hepatocarcinogenesis and that inhibition of Notch after hepatocarcinogenesis suppresses the dedifferentiation of hepatocyte.

### 2.4. Notch–Sox9 Axis Mediates Hepatocyte Dedifferentiation

**Figure 4:**
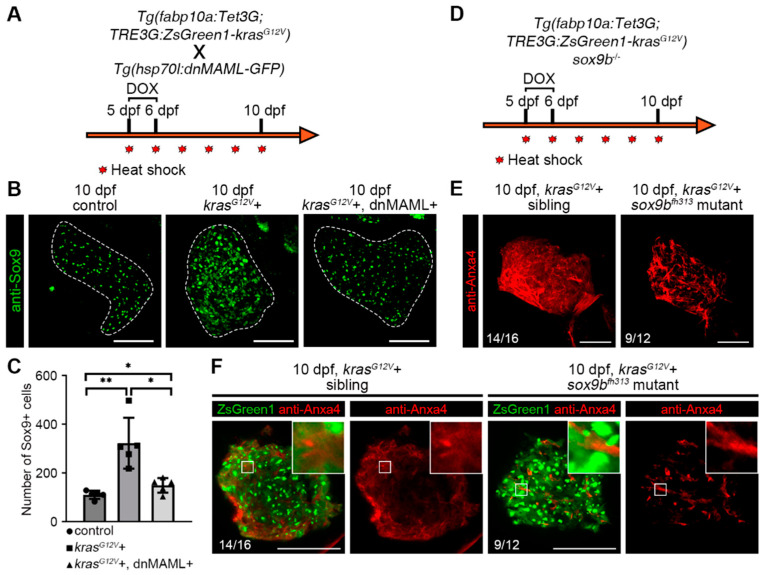
Hepatic Sox9 expression is upregulated after hepatocellular carcinoma induction, and the *sox9b^fh313^* mutant suppressed hepatocyte dedifferentiation. (**A**) *Tg(fabp10a:Tet3G;TRE3G:kras^G12V^-ZsGreen1)* with *Tg(Hsp70l:dnMAML-GFP)* double transgenic fish line treatment strategy. (**B**,**C**) Sox9 antibody staining indicated the number of Sox9+ cells in the liver at 10 dpf in the DOX-induced control (*n* = 5), *kras^G12V^*+ (*n* = 5) and *kras^G12V^*+ and dnMAML+ (*n* = 5) groups with statistical results. (**D**) *Tg(fabp10a:Tet3G;TRE3G:kras^G12V^-ZsGreen1)* treatment strategy in *sox9b^fh313^* mutant. (**E**) Three-dimensional images of Anxa4 antibody staining showed biliary duct changes in the DOX-induced *kras^G12V^*+ and *kras^G12V^*+ *sox9b^fh313^* mutants at 8 dpf and 10 dpf. (**F**) Monolayer images of Anxa4 antibody staining biliary duct changes in the DOX-induced *kras^G12V^*+ and *kras^G12V^*+ *sox9b^fh313^* mutant at 8 dpf and 10 dpf. Asterisks show significance: *—*p* < 0.05; **—*p* < 0.01. Scale bars—100 μm; error bars—S.D.

Sox9 expression in hepatocytes usually indicates that the cells are bipotential and have the ability to differentiate into both hepatocytes and biliary duct cells [37]. Previous studies have found that high Sox9 expression in hepatocellular carcinoma promotes stemness and tumorigenesis in liver cancer stem cells and is also associated with poor survival rates in patients with liver cancer [38]. The results of antibody staining of the biliary duct marker Sox9 showed that the number of Sox9-positive cells in the liver increased after hepatocarcinogenesis, and the number of Sox9-positive cells was reduced by inhibiting the Notch signaling pathway (Figure 4A–C). This is consistent with the previously reported regulation of SOX9 expression by Notch [39]. The zebrafish *sox9b^fh313^* mutant exhibited abnormal biliary duct development [40], then we induced hepatocellular carcinoma in *sox9b^fh313^* mutants and found that, similar to the inhibition of the Notch signaling pathway, the expression of biliary duct markers in hepatocytes was suppressed (Figure 4D–F). These data suggest that inhibition of the Notch signaling pathway suppressed Sox9 upregulation following hepatocarcinogenesis, and that *sox9b^fh313^* mutants also exhibited suppression of hepatocyte dedifferentiation after hepatocarcinogenesis; the Notch–Sox9 axis mediates the process of hepatocyte dedifferentiation.

### 2.5. Inhibition of Notch Signaling Ameliorated Symptoms of Liver Cancer

Proliferative HCC is often poorly differentiated and includes tumors with progenitor characteristics [41]. Fibrosis always occurred after the progression of hepatocellular carcinoma [42]. By H&E staining and Sirius Red staining in several stages, we found that the appearance of fibrosis after hepatocarcinogenesis in different stages was effectively alleviated by inhibiting the Notch signaling pathway (Figure S2A–D). Fibrosis is characterized by excess deposition of collagens, primarily type I collagen [43]. The results of the collagen I antibody staining similarly indicated that collagen fiber accumulation in the post-cancerous liver was alleviated after inhibition of Notch signaling pathway (Figure S3A–C).

**Figure 5:**
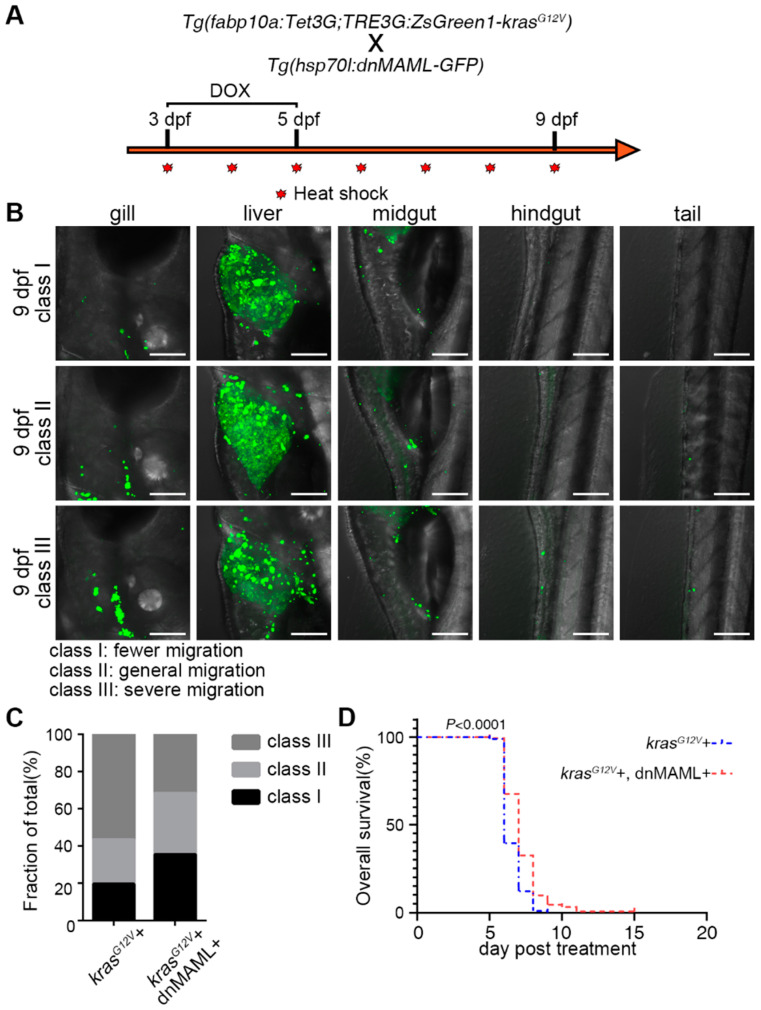
Inhibition of the Notch signaling pathway after liver cancer induction reduced cancer cell migration and improved survival. (**A**) *Tg(fabp10a:Tet3G;TRE3G:kras^G12V^ -ZsGreen1)* with *Tg(Hsp70l:dnMAML-GFP)* double transgenic fish line treatment strategy to observe the migration of cancer cells. (**B**) Zebrafish larvae were classified into three classes, I, II, and III, by the number of cancer cells outside the liver and the number of locations of metastases. (**C**) The proportion of the total number of larvae in different metastasis classes in the *kras^G12V^*+ (*n* = 46) and *kras^G12V^*+ and dnMAML+ (*n* = 130) groups at 9 dpf. (**D**) Kaplan–Meier survival curves of the DOX-induced *kras^G12V^*+ (*n* = 191) and *kras^G12V^*+ and dnMAML+ (*n* = 163) groups. *p* values for survival curves were calculated by log-rank test. *Scale bars*, 100 μm.

By inducing hepatocarcinogenesis before liver maturation, a more pronounced migration of cancer cells had been observed (Figure 5A). We classified zebrafish larvae into three classes based on the number of cancer cells outside the liver and how many locations they metastasized to (Figure 5B). The statistics of different groups of metastasis status showed that cancer cell migration after hepatocarcinogenesis was significantly reduced in the Notch-inhibited group, with a decrease in the number of severely metastasized zebrafish larvae and an increase in the number of moderately and mildly metastasized larvae (Figure 5C). This suggested that the migration of cancer cells can be prevented by inhibiting the Notch signaling pathway after hepatocarcinogenesis. Finally, the survival rate after hepatocarcinogenesis showed that inhibition of the Notch signaling pathway slightly improved the mortality caused by hepatocarcinoma induction (Figure 5D). In summary, when hepatocarcinoma occurred, inhibition of the Notch signaling pathway effectively alleviated cancer-induced fibrosis, reduced cancer cell migration, and improved survival. Taken together, inhibition of the Notch signaling pathway after hepatocarcinogenesis showed a positive effect on cancer progression.

## 3. Discussion

Hepatocellular carcinoma remains one of the leading causes of cancer death worldwide due to its high morbidity, mortality, and advanced liver dysfunction [1]. Even though there are numerous treatments available to improve the survival rate of patients with hepatocellular carcinoma, both liver transplantation and chemotherapy have several disadvantages for patients, particularly those with advanced stages of the disease [2,44]. For patients with advanced disease, systemic therapy is a more desirable treatment modality to improve patient survival. After the successful application of synergistic therapies for acute promyelocytic leukemia, synergistic therapies for HCC have also been proposed [45]. The genetic basis of most solid tumors is considerably more complex than in leukemia because it involves the cooperation of multiple oncogenic pathways. The purpose of differentiation therapy is to use drugs to induce the differentiation of cancer cells into benign cells or even normal cells [7,8]. It may be a new therapy for HCC to reduce the recurrence rate and improve the prognosis. In recent years, research on molecules and signaling pathways regulating the dedifferentiation of hepatocellular carcinoma has contributed to the development of new drugs [46]. Most solid tumors have a genetic base that requires the collaboration of numerous oncogenic pathways, making them far more complex than leukemia. Solid tumor differentiation is also more difficult to assess than leukemia, as tissue collection is difficult to repeat and in vitro cultures are usually difficult [45]. Therefore, further exploration of it in animal models may provide better assistance for future differentiation therapy in the treatment of liver cancer. Therefore, our model of *Kras^G12V^*-induced zebrafish liver cancer may provide an effective research tool for differentiation therapy.

Hepatocarcinogenesis is always accompanied by changes in the differentiation status of hepatocytes, which cause tumors to comprise heterogeneous cell populations with different differentiation states [47]. The differentiation state of cancer has been linked to its sensitivity to targeted therapy; although, the molecular mechanism behind this remains unknown [48]. We found that, after *kras^G12V^* induced hepatocarcinogenesis in zebrafish, the fate of hepatocytes and biliary duct cells in the liver was altered, and hepatocytes underwent dedifferentiation into progenitor cells with dual characteristics of liver and biliary ducts. The Notch signaling pathway is upregulated after hepatocarcinogenesis. Therefore, we tried to inhibit the dedifferentiated hepatocyte fate by inhibiting the Notch signaling pathway after hepatocarcinogenesis. We found that inhibition of the Notch signaling pathway suppressed the expression of biliary duct marker genes in hepatocytes while suppressing Sox9 upregulation due to dedifferentiation. Similarly, the dedifferentiation of hepatocytes could be inhibited in *sox9b^fh313^* mutants. Therefore, after hepatocarcinogenesis, inhibition of the Notch signaling pathway or *sox9b^fh313^* mutant suppresses dedifferentiation of hepatocytes, allowing hepatocytes to maintain their hepatocyte fate (Figure S4). Inhibition of the Notch signaling pathway also resulted in reduced liver fibrosis after hepatocarcinogenesis, a decrease in the number of migrating hepatocellular carcinoma cells, and improved survival (Figure S4).

Higher expression of *SOX9* promotes stemness and tumorigenesis in liver cancer stem cells and is also associated with poor survival [38]. Moreover, downregulation of *SOX9* decreased the invasiveness and migration of HCC [49]. As a transcription factor, *SOX9* acts as a pleiotropic factor by regulating several key signaling pathways in cancers, such as the Wnt/β-catenin, TGFβ/Smad, PI3K/AKT, and mTOR signaling pathways [38]. *SOX9* has been shown to influence cancer progression through Wnt signaling [50]. The Wnt signaling pathway is a key signaling pathway that regulates hepatocyte differentiation, and *SOX9*’s overexpression activated Wnt/β-catenin signaling through enhanced phosphorylation of GSK3β, thereby enhancing the translocation of β-catenin from the cytoplasm to the nucleus and finally enhancing the transcriptional activity of TCF1/LEF1 [50]. In addition, highly expressed *SOX9* promotes proliferation of basal cell carcinoma by directly transcriptionally regulating mTOR, which is a crossover pathway between hedgehog signaling and PI3K/AKT/mTOR pathways [51]. A study found that mTORC1 signaling controls liver regeneration by regulating the dedifferentiation of biliary epithelial cells [52]. Therefore, we speculate that, in this zebrafish liver cancer model, Sox9 may regulate hepatocyte dedifferentiation through the Wnt–mTOR signaling pathway. Novel specific *SOX9* inhibitors or compounds that attenuate *SOX9* expression in *SOX9*-driven tumors will be important for future personalized anticancer treatment strategies [53].

Previous studies found that Notch signaling in hepatocellular carcinoma promotes cell stemness and poor differentiation. Liver cancer stem cells originated from either hepatocytes dedifferentiation or differentiation arresting of liver normal stem cells. In a previous study, it was found that the inhibition of Notch promotes the differentiation of malignantly transformed hepatic progenitor cells [54]. We propose that, in our model, hepatocellular carcinoma stem cells arise mainly derived from hepatocyte dedifferentiation after hepatocarcinogenesis, rather than hepatocellular carcinoma stem cells self-replicating without differentiation. This is because our model is of hepatocellular carcinoma resulting from specific expression of oncogenes in mature hepatocytes in zebrafish, and previous studies used cell lines to conclude this [55]. Additionally, we observed that hepatocytes subsequently expressed hepatic progenitor cell markers after hepatocarcinogenesis. Considering the timing, hepatocyte dedifferentiation should be the main contribution of hepatic progenitor cells at this time. Of course, this does not exclude the contribution of hepatocellular carcinoma stem cells self-replicating without differentiation to them. The specific contribution of the two ways in hepatocarcinogenesis still needs to be further investigated. In summary, this study reveals the role of Notch in hepatocyte dedifferentiation through downregulation of Sox9 in *kras^G12V^*-induced hepatocellular carcinoma, providing a potential target for clinical studies of HCC. In addition, this model provides a powerful tool for further screening of differentiated therapeutic agents.

In conclusion, we found that hepatocyte dedifferentiation is closely associated with hepatocellular carcinoma progression after hepatocarcinogenesis, and inhibition of the Notch signaling pathway allows hepatocytes to maintain their hepatocyte fate. Whether the same findings hold in more heterogeneous hepatocellular carcinoma models needs to be further investigated. The specific mechanisms by which Notch specifically regulates hepatocyte dedifferentiation into hepatocellular carcinoma cells after hepatocarcinogenesis require further study. Further single-cell sequencing may make an essential contribution to unraveling this question.

## 4. Materials and Methods

### 4.1. Ethical Statement

All experimental protocols were approved by the Institute of Developmental Biology and Regenerative Medicine, Southwest University (Chongqing, China), and methods were performed according to approved guidelines. The zebrafish facility and studies were approved by the Institutional Review Board of Southwest University (Chongqing, China). Zebrafish were housed by the Guide for Laboratory Animal Welfare of the Ministry of Science and Technology of the People’s Republic of China (2006) and the protocols of the Institutional Animal Care and Use Committee of Southwest University (2007).

### 4.2. Zebrafish Lineage

Zebrafish (*Danio rerio*) AB strain-derived *Tg(fabp10a:Dendra2-NTR)^cq1^* were used as control [29], for the induction of liver cancer by *Tg(fabp10a:Tet3G;TRE3G:kras^G12V^-ZsGreen1)^cq136^* from AB strain injected with both *pfabp10a:Tet3G* and *pTRE3G-BI:kras^G12V^-ZsGreen1*. Plasmids were constructed, and *Tg(Hsp70l:dnMAML-GFP)* was used for inhibition of Notch signaling. These zebrafish lines were raised under standard conditions and embryos/larvae used for experiments were treated with 0.003% PTU (Sigma) starting from 24 hpf.

### 4.3. Whole-Mount In Situ Hybridization

Whole-mount in situ hybridization (WISH) was performed according to the previously described method [52]. The antisense probes for *hhex*, *foxa3*, *sox9b*, *notch1a*, *notch1b*, *notch2*, *notch3*, and *her15* were used.

### 4.4. Fluorescent In Situ Hybridization Coupled with Antibody Staining Assays

Fluorescent in situ hybridization (FISH) coupled with antibody staining assays was performed according to the previously described method [56,57]. The antisense probes for *sox9b* and *cp* were used.

### 4.5. Antibody Staining

Antibody staining was performed as previously described [58,59]. The following antibodies were used: antibody to Dendra2 (1:1000; AB821, Evrogen, Moscow, Russia), anti-CDH1 (1:1000; 610181, BD Biosciences), anti-Anxa4 (2F11) (1:1000; ab71826, Abcam, Cambridge, MA, USA), anti-SOX9 (1:500; A5080, Bimake), Anti-Collagen I (1:500; ab23730, Abcam, Cambridge, MA, USA), and anti-PCNA (1:1000; SAB2701819, Sigma).

### 4.6. Generation of Transgenic Lines for Induction Experiments

The *fabp10a* promoter was replaced into the modified *pEF1a:Tet3G* (Cat. 631342, Clontech) vector. The cDNA of full-length Zebrafish *kras* was amplified by PrimeSTAR HS DNA polymerase (Takara) and point mutated to obtain *kras^G12V^*, cloned into the modified *pTRE3G-BI:ZsGreen1* (Cat.631342, Clontech) vector; and TRE3G-BI is a bidirectional version of TRE3G promoter that allows for simultaneous, equivalent, and inducible expression of two transgenes. Both plasmids were injected simultaneously with *I-Sce*I enzyme into AB strain embryos to generate transgenic line *Tg(fabp10a:Tet3G;TRE3G-BI:kras^G12V^-BI-ZsGreen1)^cq136^* (referred to as *Tg(fabp10a:Tet3G;TRE3G:kras^G12V^-ZsGreen1)^cq136^*) as previously described [59,60]. Positive embryos showed expression of Cerulean in the eyes of the offspring.

### 4.7. DOX Treatment and Heat Shock

DOX (doxycycline, A600889, Sangon) at a final concentration of 40μg/mL was added to egg water at 5 dpf for activation of the Tet-On system and controls without DOX. To induce dnMAML-GFP overexpression in *Tg(Hsp70l:dnMAML-GFP)*, larvae were placed in egg water and then incubated in a 38.5 °C water bath for 30 min from 5 dpf to 10 dpf, once a day.

### 4.8. Paraffin Sectioning and Staining

Paraformaldehyde-fixed livers were embedded in paraffin. Sections (7 μm) were stained using hematoxylin and eosin (H&E) to examine cell and tissue morphology as previously described [29]. Sections (7 μm) were stained using Sirius Red (BP-DL030, Sbjbio life sciences, Nanjing) staining to detect tissue fibrosis morphology, as previously described [61]. To maintain uniformity between samples, images taken at multiple depths from 1.0 mm to 3.0 mm were used as a reference between liver sections to determine the depth of sections.

### 4.9. Percent Fibrosis Statistics

All H&E staining and Sirius Red staining were separated the eosin staining and Sirius Red staining by the color deconvolution plugin in of ImageJ software and calculating the area. Fibrosis calculations of H&E staining and Sirius Red staining were performed according to previous reports [62,63].

### 4.10. Data Collection and Analysis

All images were taken on a SteREO DiscoveryV20 microscope (Carl Zeiss, Germany), a Zeiss Axio Image Z1 (Carl Zeiss), and an LSM780 confocal microscope (Carl Zeiss). The intensities and areas of the fluorescence images were measured using ImageJ. All figures, labels, arrows, scale bars, and outlines were drawn using the Adobe Photoshop software. Statistical analysis was performed with GraphPad Prism 8. Statistical significance was determined using the unpaired two-tailed Student’s t-test. *p* values for survival curves were calculated by log-rank test. The changes of each data point are expressed as mean ± S.D.

## Data Availability

Data are available from the corresponding author upon reasonable request.

## Supplementary Materials

The following supporting information can be downloaded at: https://www.mdpi.com/article/10.3390/ijms23094705/s1.
