# Supplementary material for: Notch–Sox9 Axis Mediates Hepatocyte Dedifferentiation in KrasG12V-Induced Zebrafish Hepatocellular Carcinoma
Source: Int J Mol Sci. 2022 Apr 24;23(9):4705. doi: 10.3390/ijms23094705 (PMC9103821; doi:10.3390/ijms23094705)
Supplement: Supplementary file 1 [file ijms-23-04705-s001.zip › ijms-1671876-supplementary.pdf]

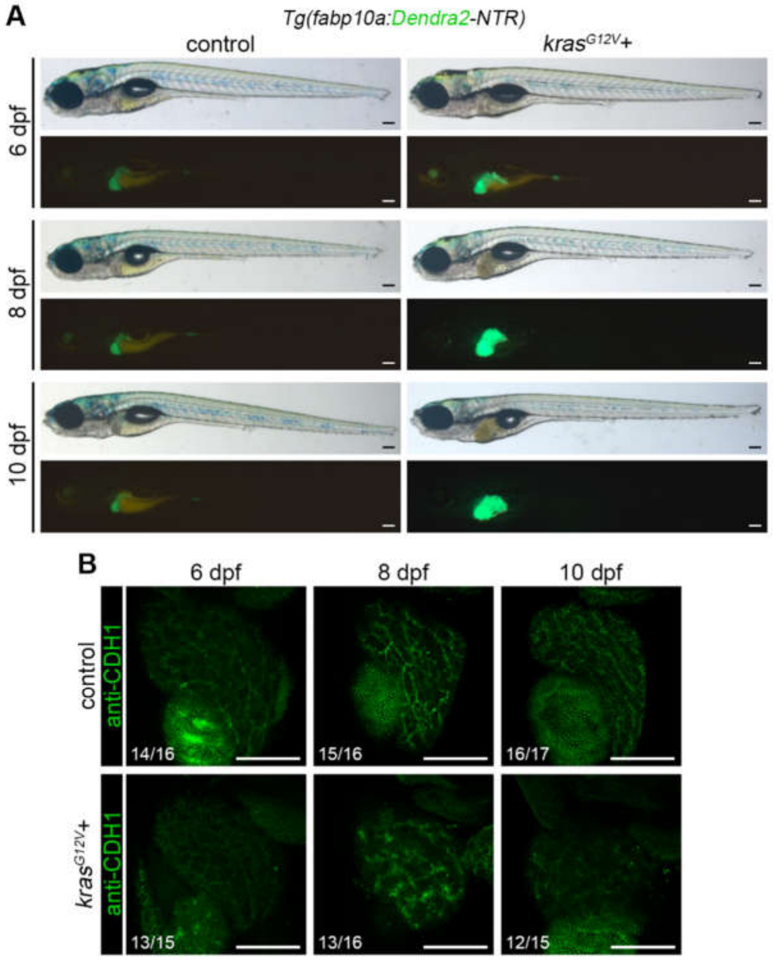


**Figure S1.** DOX-induced liver enlargement after *kras*$^{G12V}$ overexpression with no other physical abnormalities and biliary duct activation.

(**A**) Zebrafish liver after DOX activation in somatic microscopy, from 6 dpf to 10 dpf, relative to the control group, zebrafish in the *kras*$^{G12V}$+ group maintained normal body and progressively larger liver. (**B**) Results of CDH1 antibody staining in the liver of zebrafish in the DOX-induced control, *kras*$^{G12V}$+ groups at 6 dpf, 8 dpf, 10 dpf. *Scale bars*, 100 μm.

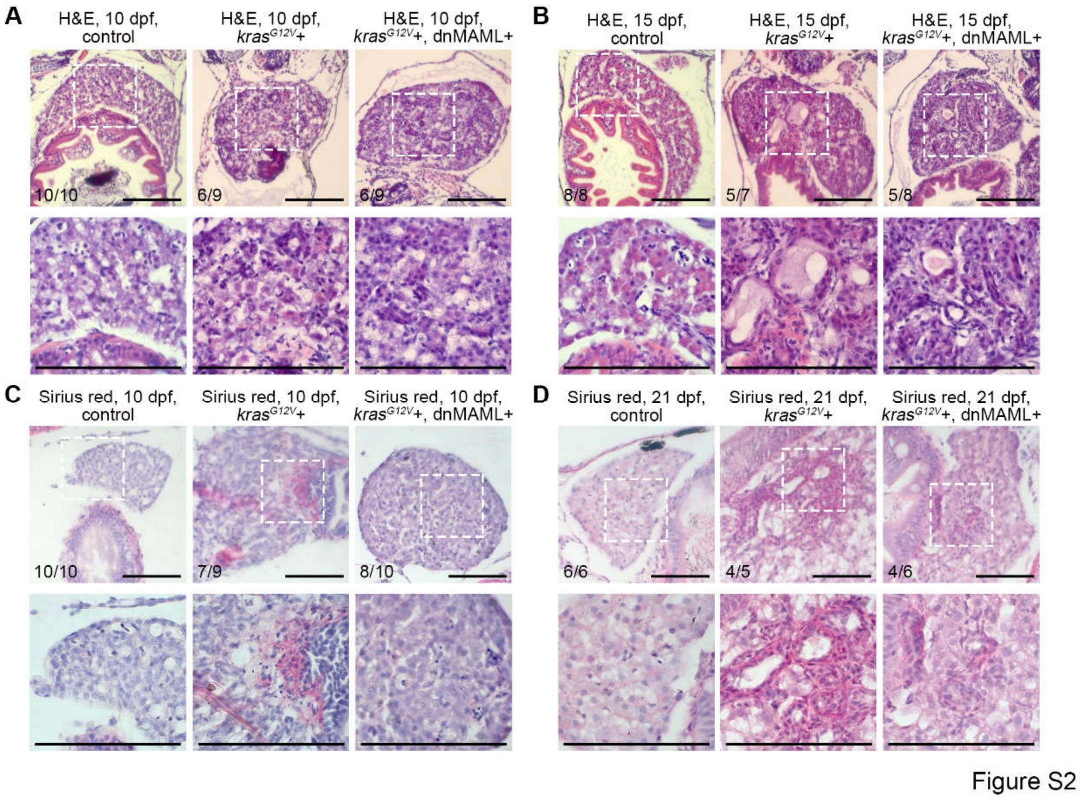


**Figure S2.** Inhibition of Notch after hepatocellular carcinoma induction alleviated liver fibrosis.

(**A**,**B**) H&E staining showed the morphology of liver in DOX-induced control, *kras*$^{G12V}$+ and *kras*$^{G12V}$+ & dnMAML+ groups at 10 dpf,15 dpf. (**C**,**D**) Sirius red staining showed liver fibrosis status in the DOX-induced control, *kras*$^{G12V}$+ and *kras*$^{G12V}$+ & dnMAML+ groups at 10 dpf, 21 dpf. *Scale bars*, 100 μm.

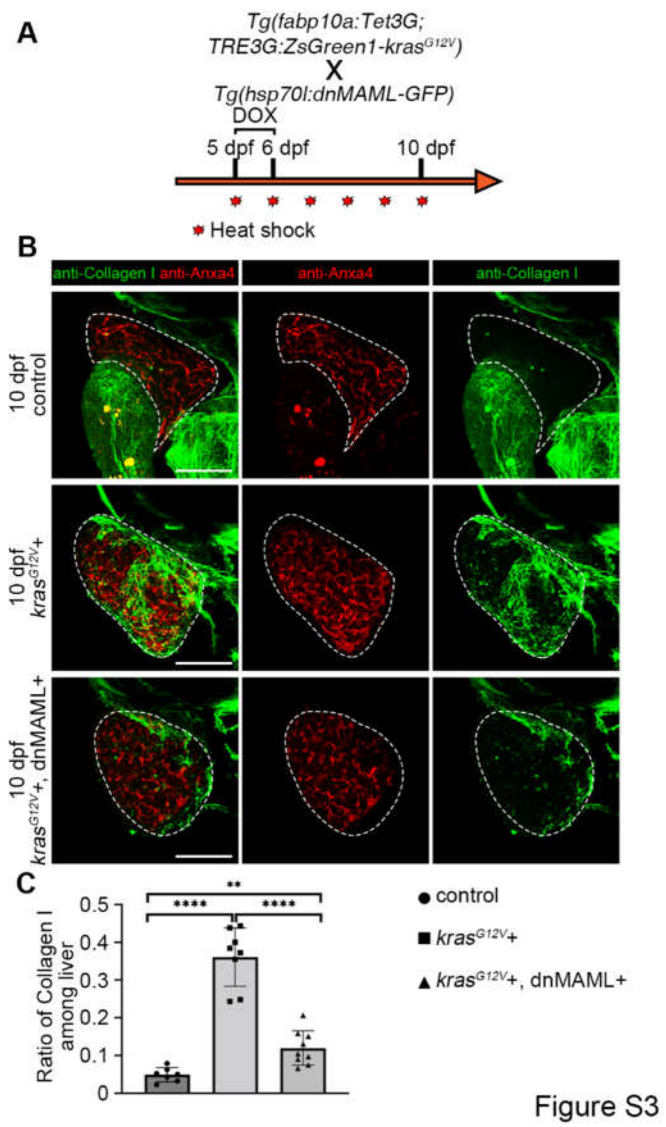


**Figure S3.** Inhibition of Notch after hepatocellular carcinoma induction alleviated collagen accumulation in the liver.

(**A**) *Tg(fabp10a:Tet3G;TRE3G:kras$^{G12V}$-ZsGreen1)* with *Tg(Hsp70l:dnMAML-GFP)* double transgenic fish line treatment strategy. (**B**) 3D images of Collagen I antibody staining showed collagen accumulation in the liver of the DOX-induced control, *kras$^{G12V}$*+ and *kras$^{G12V}$*+ & dnMAML+ groups at 10 dpf. (**C**) Statistics of the percentage of Collagen I antibody staining in the liver region in the DOX-induced control, *kras$^{G12V}$*+ and *kras$^{G12V}$*+ & dnMAML+ groups.

*Asterisks* show significance: **, $p < 0.01$; ****, $p < 0.0001$. *Scale bars*, 100 μm; *error bars*, S.D.

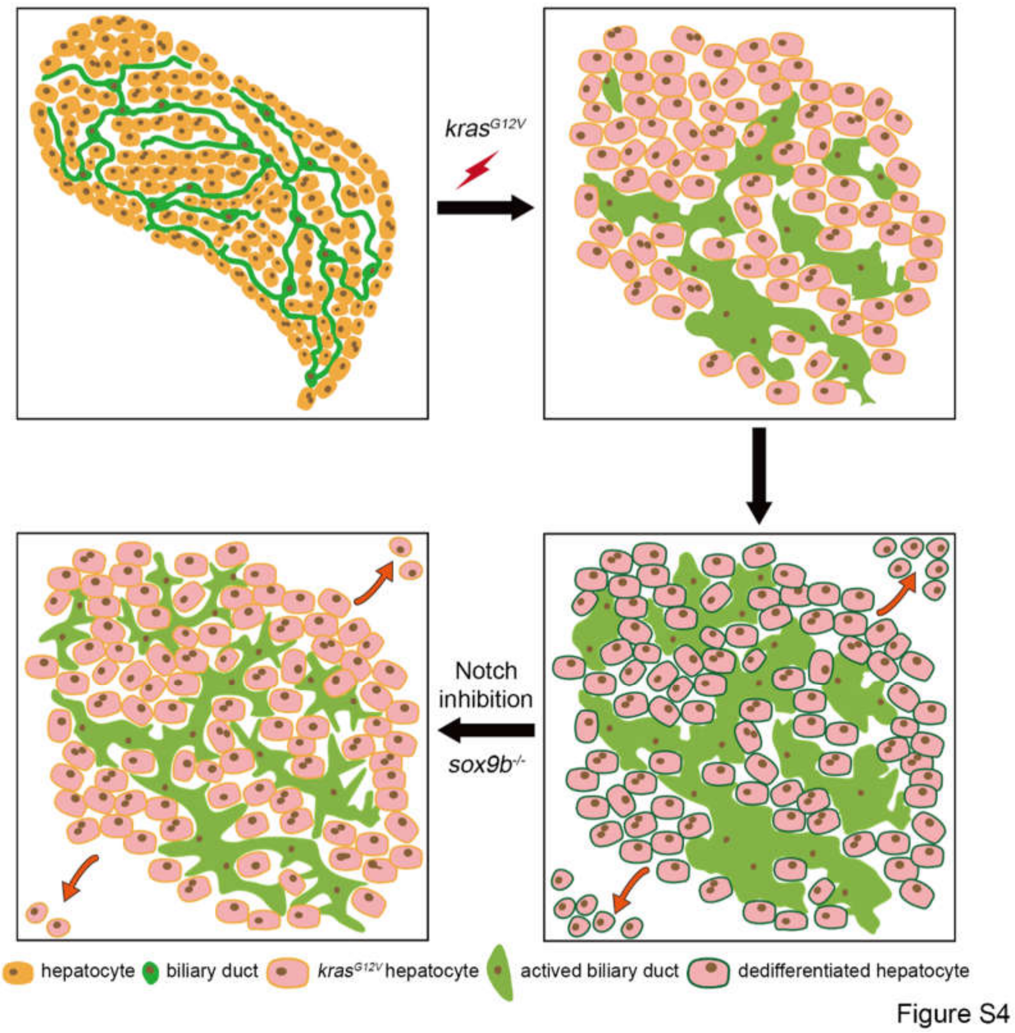


**Figure S4.** *kras*$^{G12V}$-induced hepatocarcinogenesis in zebrafish liver, and inhibition of Notch signaling suppresses hepatocyte dedifferentiation after hepatocarcinogenesis.
